# Inlay Dynamic Anterior Stabilization of the Shoulder With Long Head Biceps Tendon Using All‐Suture Anchor

**DOI:** 10.1002/atn2.70262

**Published:** 2026-09-22

**Authors:** Ricardo Mendes, Marcelo Mello, José Carlos Garcia Jr, Airthon Correia, Matheus Barcelos, Álvaro Motta, Hilton Lutfi

**Affiliations:** ^1^ NAEON Shoulder and Elbow Institute and Sports Medicine Institute São Paulo Brazil; ^2^ NAEON Institute São Paulo Brazil

## Abstract

Dynamic anterior stabilization using the long head of the biceps tendon (LHBT) has emerged as a soft‐tissue alternative for treating anterior shoulder instability in the setting of subcritical glenoid bone loss. This Technical Note describes an inlay dynamic anterior stabilization technique using soft‐tissue anchors designed to enhance tendon‐to‐bone healing and optimize the length‐tension relation of the transferred LHBT. The procedure combines a modified Bankart repair with trans‐subscapularis LHBT transfer and fixation within a 5‐mm anterior‐inferior glenoid bone tunnel using inlay soft anchors and Krakow suturing. This construct reproduces sling and hammock stabilizing effects while preserving external rotation and avoiding bone grafting. The technique allows intraoperative adaptability when labral tissue is inadequate for isolated Bankart repair and does not preclude future bone‐block procedures if required. Inlay dynamic anterior stabilization with LHBT is a reproducible and cost‐effective option for selected patients with anterior instability and glenoid bone loss less than 20%.

## Graphical abstract

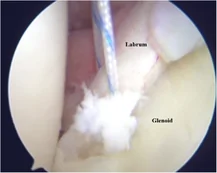

**VIDEO 1:** Inlay Dynamic Anterior Stabilization (DAS) using the long head of the biceps tendon and an all‐suture anchor. This technique combines arthroscopic Bankart repair with an intra‐articular inlay fixation of the long head of the biceps tendon inside a 20‐mm glenoid tunnel, increasing the tendon‐to‐bone contact area while preserving the native anatomy. The goal is to enhance anterior shoulder stability in patients with subcritical glenoid bone loss, providing a dynamic sling effect together with the capsulolabral repair. Video content can be viewed at https://doi.org/10.1002/atn2.70262.

Anterior shoulder instability remains a significant challenge for orthopedic surgeons. Multiple studies have shown that isolated labral repair in the presence of bone loss is associated with high failure rates, particularly in young patients and athletes. Over the years, various predictive tools have been developed to assess the risk of failure after a Bankart procedure and to assist in surgical decision‐making.

One of the most widely adopted tools is the *Instability Severity Index Score*, introduced by Balg and Boileau, which incorporates patient‐ and lesion‐specific factors to guide the choice between arthroscopic Bankart repair and bone augmentation procedures.^1^, ^2^

Notably, when the Instability Severity Index Score is 7 or higher, the recurrence rate after arthroscopic Bankart repair has been reported to reach up to 70%.^3^ Phadnis and colleagues later observed that scores of 4 or higher on the Instability Severity Index Score already showed unfavorable results (with a 13.5% failure rate), suggesting it as a new cutoff point.^4^

The optimal surgical approach for anterior glenohumeral instability associated with limited (0%‐13.5%) to subcritical (13.5%‐25%) glenoid bone loss (GBL) remains a subject of ongoing debate.^5^

Although bony transfer procedures have traditionally been recommended for GBL between 20% and 25%,^1^ there is increasing consensus that isolated Bankart repair is contraindicated in cases involving GBL greater than 21% to 25% and/or the presence of off‐track Hill‐Sachs lesions.^6^, ^7^, ^8^ In such scenarios, bone augmentation techniques, including the Bristow and Latarjet procedures, are considered more reliable and biomechanically stable.^6^, ^9^

However, more recent studies have suggested that even GBL as low as 13.5% may be associated with unfavorable outcomes, including reduced functional scores—particularly among active individuals and overhead athletes.^5^

In another investigation, a critical bone loss threshold of 17.3% was identified as a predictive factor for failure.^10^ These findings highlight the clinical uncertainty surrounding the optimal management of anterior shoulder instability in active patients with subcritical (<20%) GBL, where the decision between isolated Bankart repair and bone augmentation procedures remains controversial and individualized. Dynamic anterior stabilization (DAS) using the long head of the biceps (LHB) tendon has recently emerged as a novel technique for the treatment of anterior shoulder instability.^11^, ^12^

This procedure offers several potential advantages over the traditional Latarjet technique, including reduced morbidity and the ability to simultaneously address associated SLAP lesions.^4^ Although the long head of the biceps tendon (LHBT) in glenohumeral stability has historically been considered limited,^13^, ^14^ more recent investigations have questioned its functional significance altogether. Some authors have proposed that the LHBT may represent a vestigial structure, lacking a biomechanically relevant axis for shoulder stabilization in bipedal species, unlike its function in quadrupeds.^15^

Transposition of the LHB tendon through the subscapularis produces both the “hammock” and “sling” effects,^13^ potentially enhancing shoulder stability to a greater extent than the traditional Latarjet procedure. The hammock effect occurs in the early degrees of abduction, where the LHB depresses the inferior third of the subscapularis during the first 30° of abduction and external rotation. The sling effect becomes predominant beyond 30°, acting as a dynamic restraint to anterior humeral head translation as abduction and external rotation increase.

These combined stabilization mechanisms, when performed in conjunction with a Bankart repair, may yield superior outcomes in patients within the so‐called “gray zone” of GBL.

Cadaveric studies have shown that a Bankart repair performed in the presence of 15% GBL may result in restricted external rotation, a limitation also observed with the Latarjet procedure.^16^, ^17^, ^18^ Notably, this restriction is not observed with the DAS technique.^19^

In models of anterior instability with 10% and 20% GBL, DAS significantly reduced anterior glenohumeral translation when compared with isolated Bankart repair.^17^ Furthermore, in 13% GBL models, DAS showed 16.3% greater biomechanical strength than Bankart repair alone.^3^

Various fixation methods for the LHB tendon have been described, including biotenodesis screws,^11^, ^20^ suture anchors,^7^ and cortical buttons.^21^, ^22^

The aim of this study is to describe an inlay DAS technique procedure using inlay suture anchors for LHB fixation.

In contrast to the traditional onlay technique,^23^ we advocate for the creation of a tendon‐specific bone socket, matched to the diameter of the LHB tendon. This approach allows for optimized tensioning of the LHB sling, increased tendon‐to‐bone contact area, and potentially enhanced biological healing and greater mechanical strength of the fixation.

## SURGICAL TECNIQUE

The patient is placed in the beach‐chair position under general anesthesia combined with an interscalene brachial plexus block.

A standard diagnostic arthroscopy is performed through the posterior portal. A working anteroinferolateral portal is then established, providing access to the rotator interval and the bicipital groove. A cannula (Zimmer Biomet, Warsaw, IN) is introduced through this portal to facilitate instrumentation.

**FIGURE 1:**
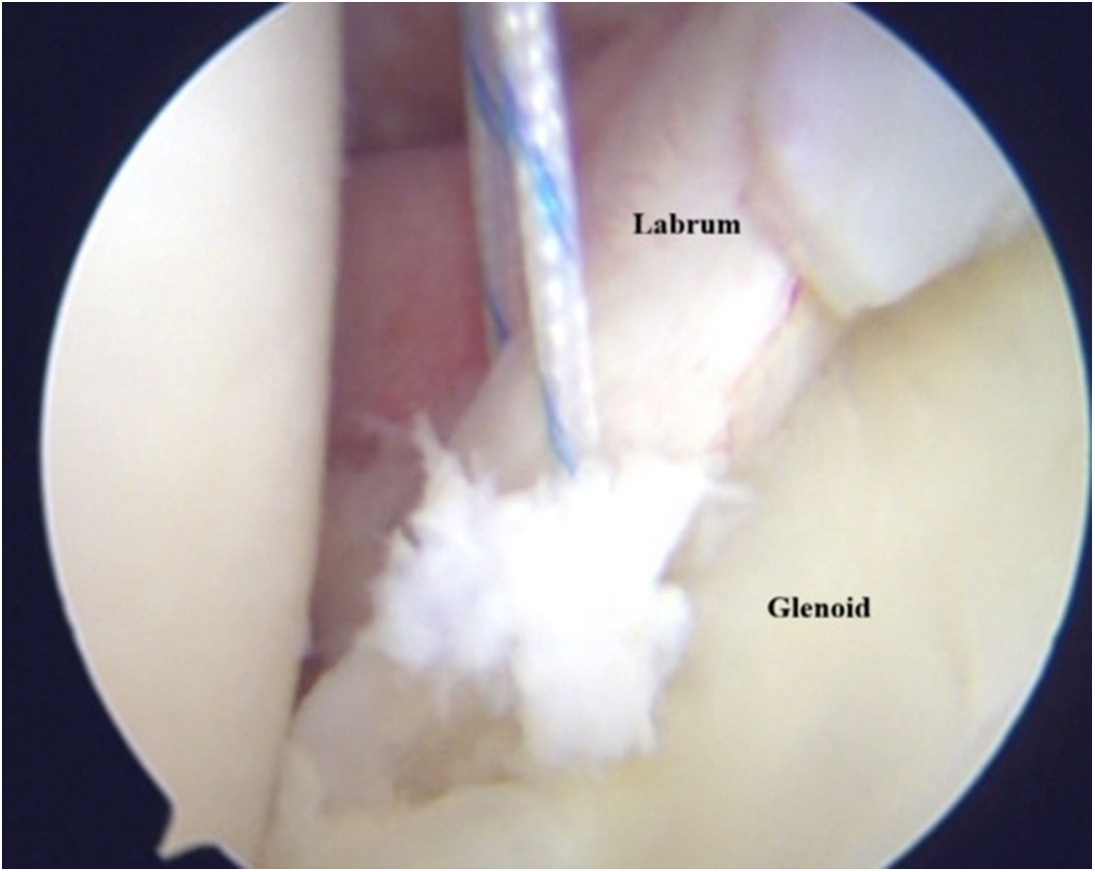
Patient in the beach‐chair position, posterior viewing portal in the left shoulder. Through the anterior portal, 1.5‐mm anchor (JuggerKnot, Zimmer Biomet, Warsaw, IN) was inserted at the 5‐o'clock position of the right glenoid to repair the anteroinferior capsulolabral complex.

Complete labral mobilization and glenoid neck decortication are performed, exposing the anterior glenoid rim down to subchondral bone to prepare the bed for anchor placement and capsulolabral repair. A 1.5‐mm soft‐tissue all‐suture anchor (JuggerKnot, Zimmer Biomet, Warsaw, IN) is inserted at the anteroinferior glenoid margin (5‐o'clock position on a right shoulder), allowing an anatomical reconstruction of the labrum and capsule, a variation of the classic Bankart repair (Figure 1).

This capsulolabral reinforcement, when combined with the dynamic stabilizing effect of the LHB, contributes to anterior glenohumeral stability by restoring tension to the inferior glenohumeral ligament complex.

Using the same anteroinferolateral portal, a bipolar electrocautery device (Zimmer Biomet, Warsaw, IN) was employed to release the LHBT along the bicipital groove, achieving transverse humeral ligament rupture and complete mobilization of the tendon.

Through this portal, a spinal needle is advanced under direct visualization to pierce the subscapularis muscle toward the glenoid neck between the 3‐ and 4‐o'clock positions (right shoulder).

**FIGURE 2:**
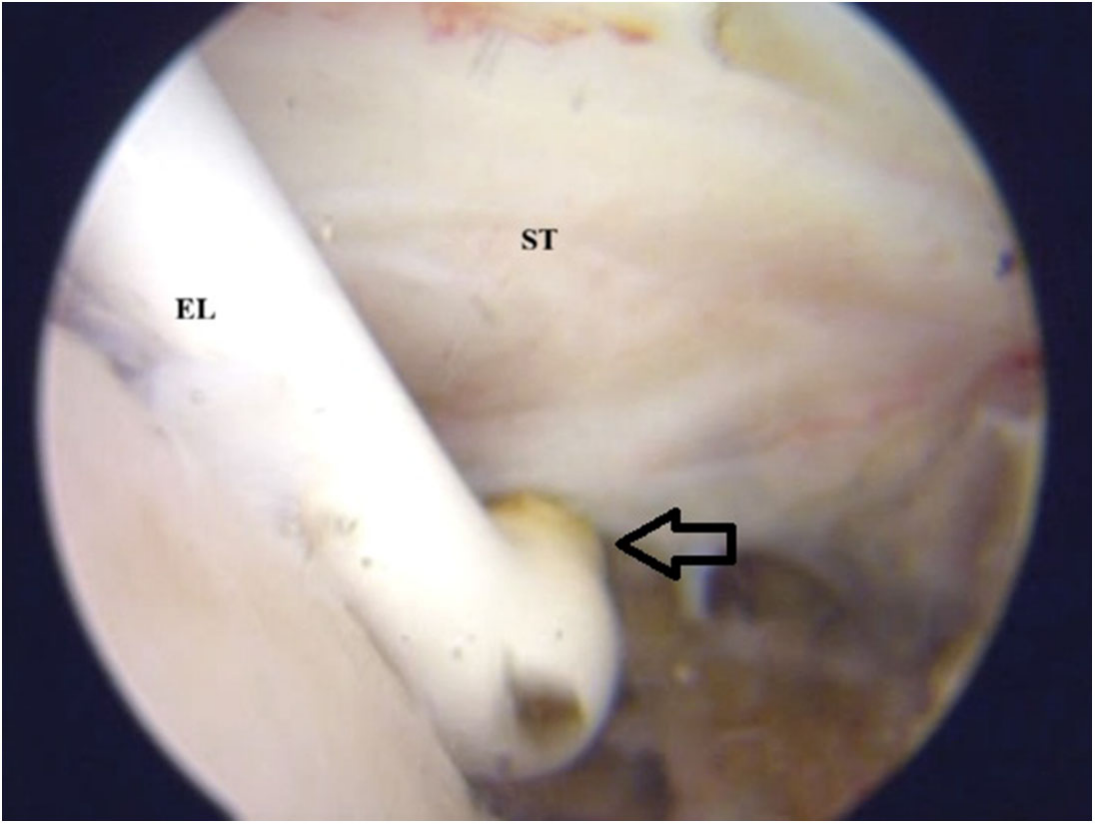
Patient in the beach‐chair position, posterior viewing portal in the left shoulder. EL (Zimmer Biomet, Warsaw, IN) (black arrow), performing a longitudinal incision in the inferior two‐thirds of the ST, following the direction of its fibers. (EL, electrocautery; ST, subscapularis tendon.)

A longitudinal, controlled incision is then performed through the center of the subscapularis tendon using a Kelly clamp, and the release is completed with electrocautery (Zimmer Biomet, Warsaw, IN), allowing safe passage of the biceps tendon through the muscle belly (Figure 2).

An anterolateral portal is established, and using a Kocher clamp introduced through this portal, the LHBT is grasped superior to the subscapularis. An arthroscopic tenotomy is then performed, followed by exteriorization of the tendon through the same portal.

An anterior cruciate ligament (drill guide system, Zimmer Biomet, Warsaw, IN) is then positioned at the anteroinferior glenoid rim, between the 3‐ and 4‐o'clock positions (right shoulder), near the osteochondral junction. It is critical that the tunnel is drilled within 5 mm of the glenoid edge to preserve stability and avoid medial malpositioning of the graft.

**FIGURE 3:**
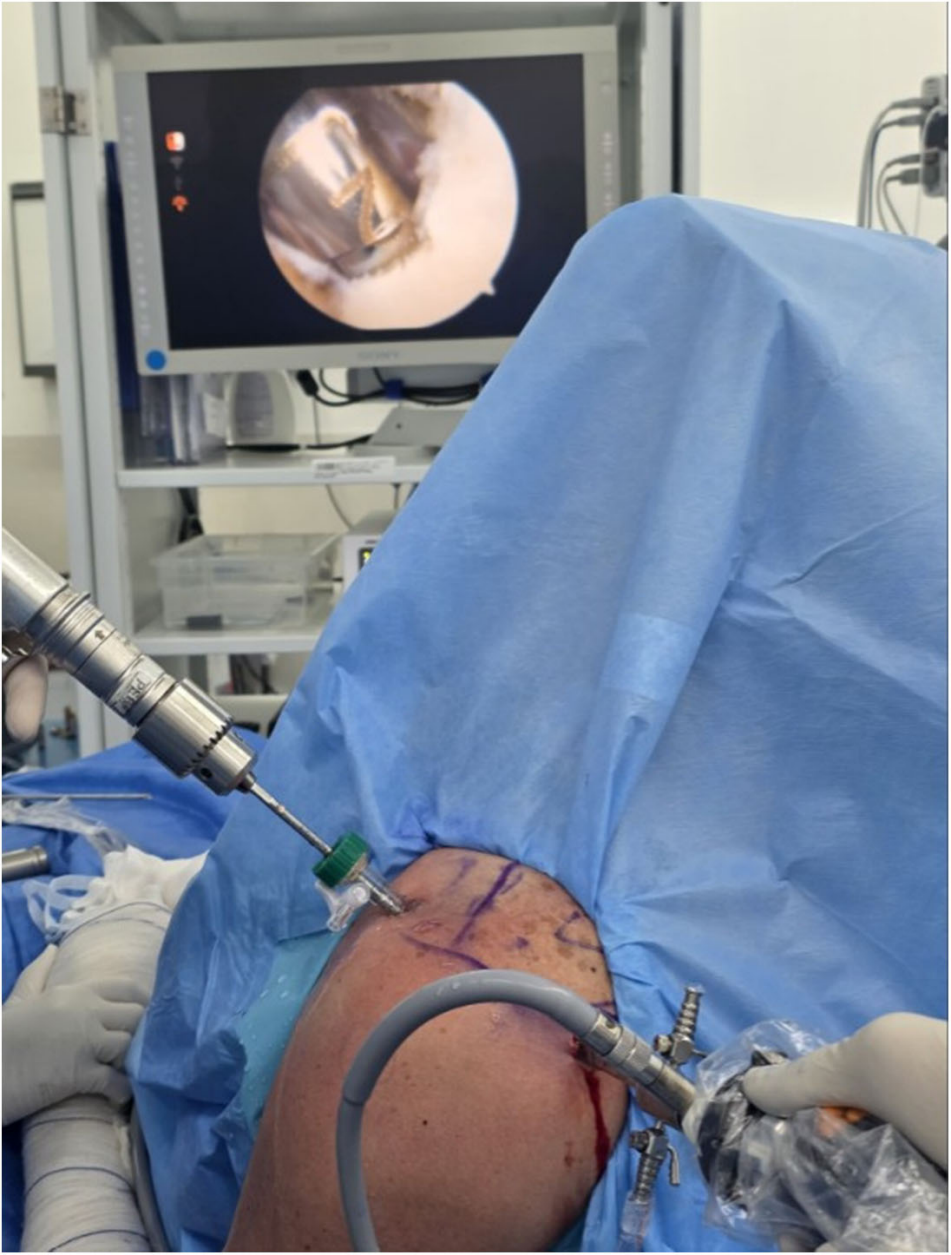
Patient in the beach‐chair position, posterior viewing portal in the left shoulder. Through the anteroinferior portal through the subscapular open wedge, a 5‐mm drill (ACL drill guide system by Zimmer Biomet, Warsaw, IN) was used to create a 2‐cm deep perforation at the 3‐ to 4‐o'clock position of the glenoid. (ACL, anterior cruciate ligament.)

**FIGURE 4:**
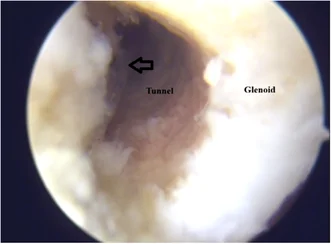
Patient in the beach‐chair position, through the anterolateral portal view in the left shoulder, a 5‐mm safety margin was observed during drilling, with the anchor positioned at a deeper level (black arrow, suture anchor JuggerKnot, Zimmer Biomet, Warsaw, IN).

**FIGURE 5:**
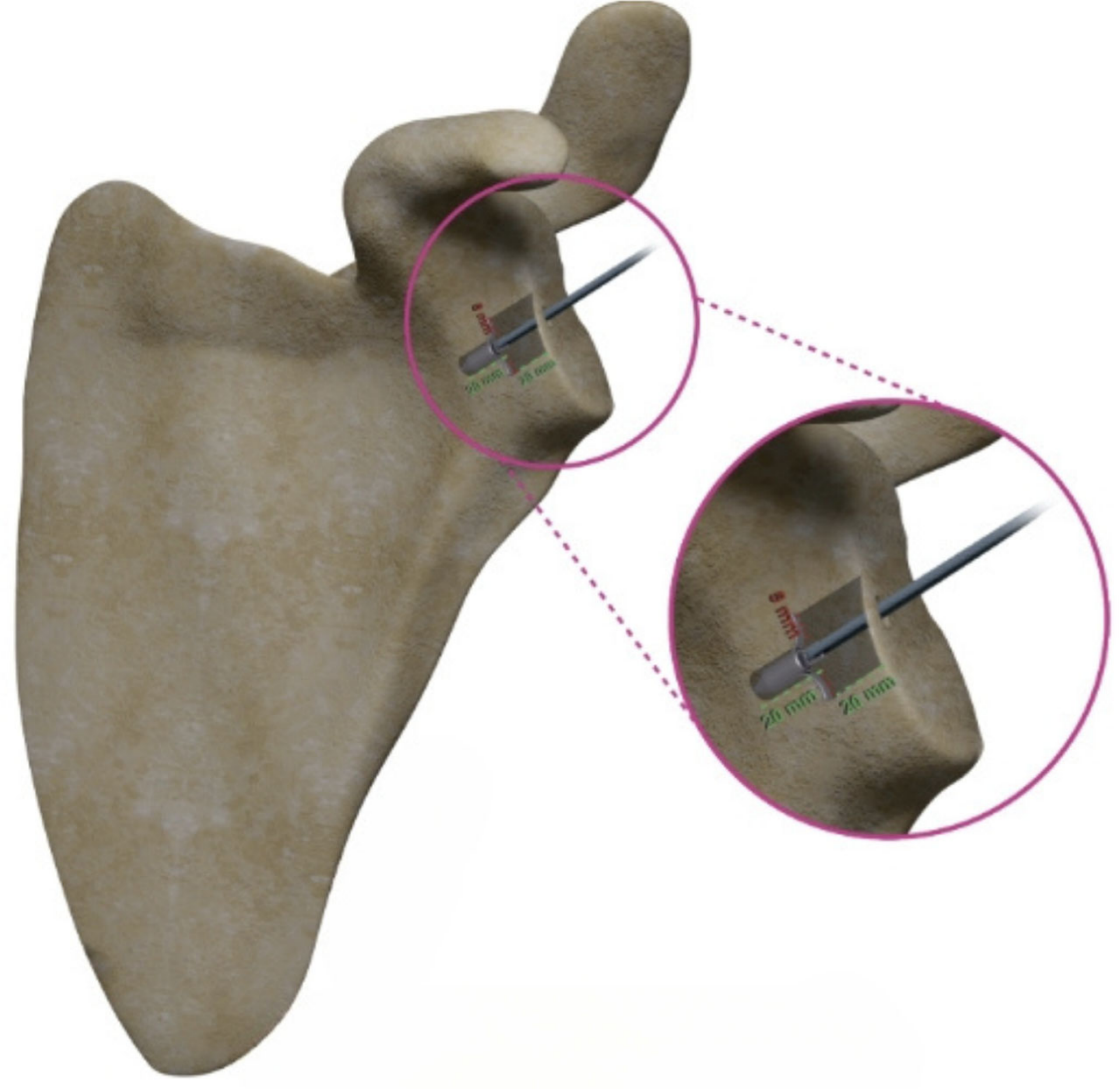
Illustrative image showing a dashed line representing the 2‐cm tunnel depth, with a red dashed overlay indicating the 5‐mm tunnel width, ensuring a minimum 5‐mm margin from the glenoid rim and the correct anchor placement.

Using a 5‐mm drill, a bone tunnel approximately 20 mm in depth is created. After tunnel preparation, the instability anchor guide is inserted into the predrilled hole, and a soft‐tissue anchor is deployed at a depth of 15 mm, ensuring secure fixation within the glenoid neck (Figures 3, 4, 5).

**FIGURE 6:**
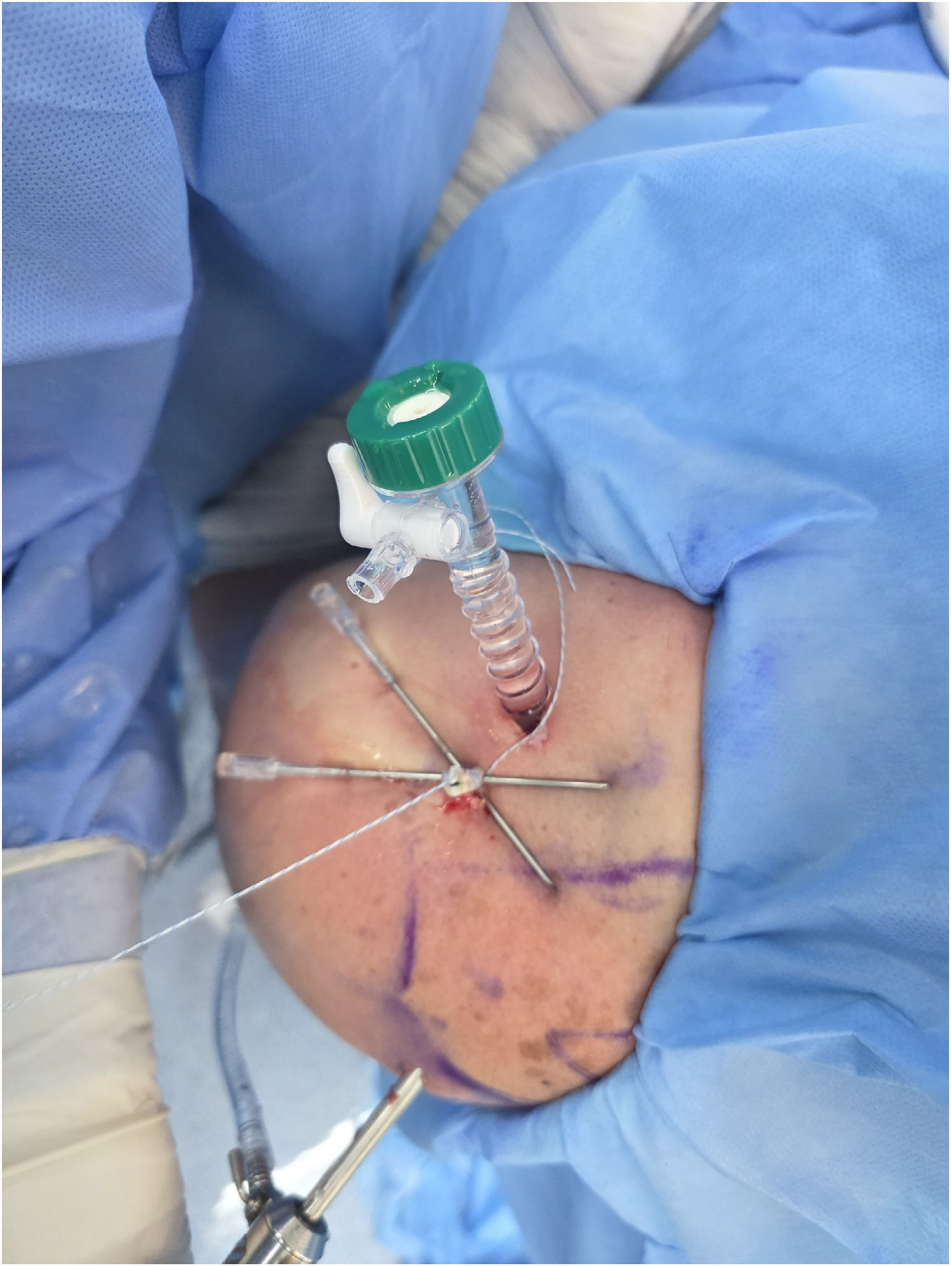
In the left shoulder, with outside, a Krakow suture was performed using one of the anchor sutures (JuggerKnot, Zimmer Biomet, Warsaw, IN).

**FIGURE 7:**
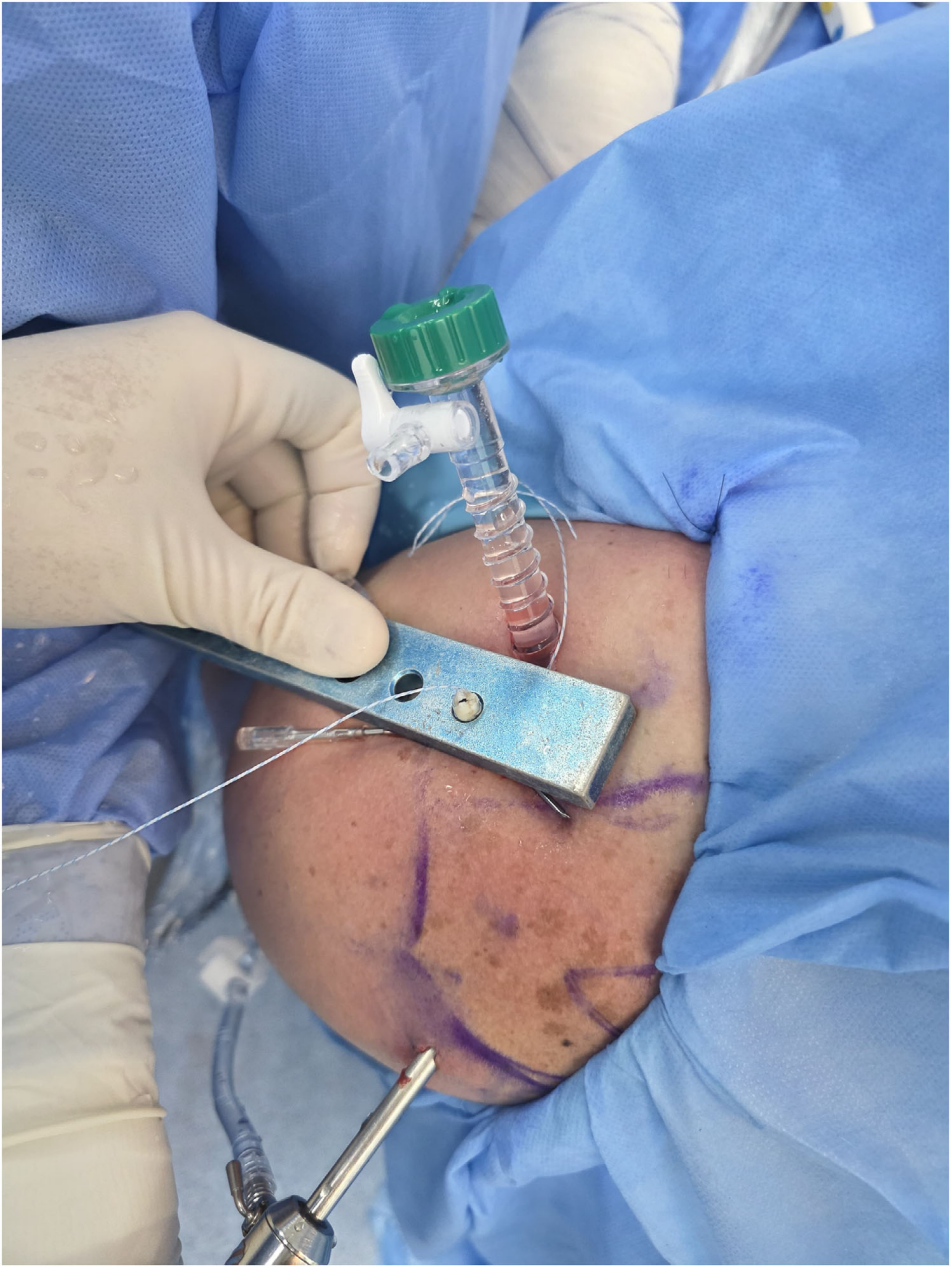
After suturing, the length of the long head of the biceps tendon was measured and found to be compatible with the 5‐mm tunnel.

The sutures from the anchor (JuggerKnot, Zimmer Biomet, Warsaw, IN) are then retrieved through the same portal. The LHBT is secured extra‐articularly using a Krakow suture technique with a single suture strand from the anchor, maintaining 20 mm of tendon length, consistent with the measured tunnel depth. The tendon diameter is measured, typically ranging from 5 to 6 mm, to ensure compatibility with the previously drilled bone tunnel. After suturing, the tendon diameter is reassessed to confirm appropriate sizing for inlay fixation (Figures 6 and 7).

**FIGURE 8:**
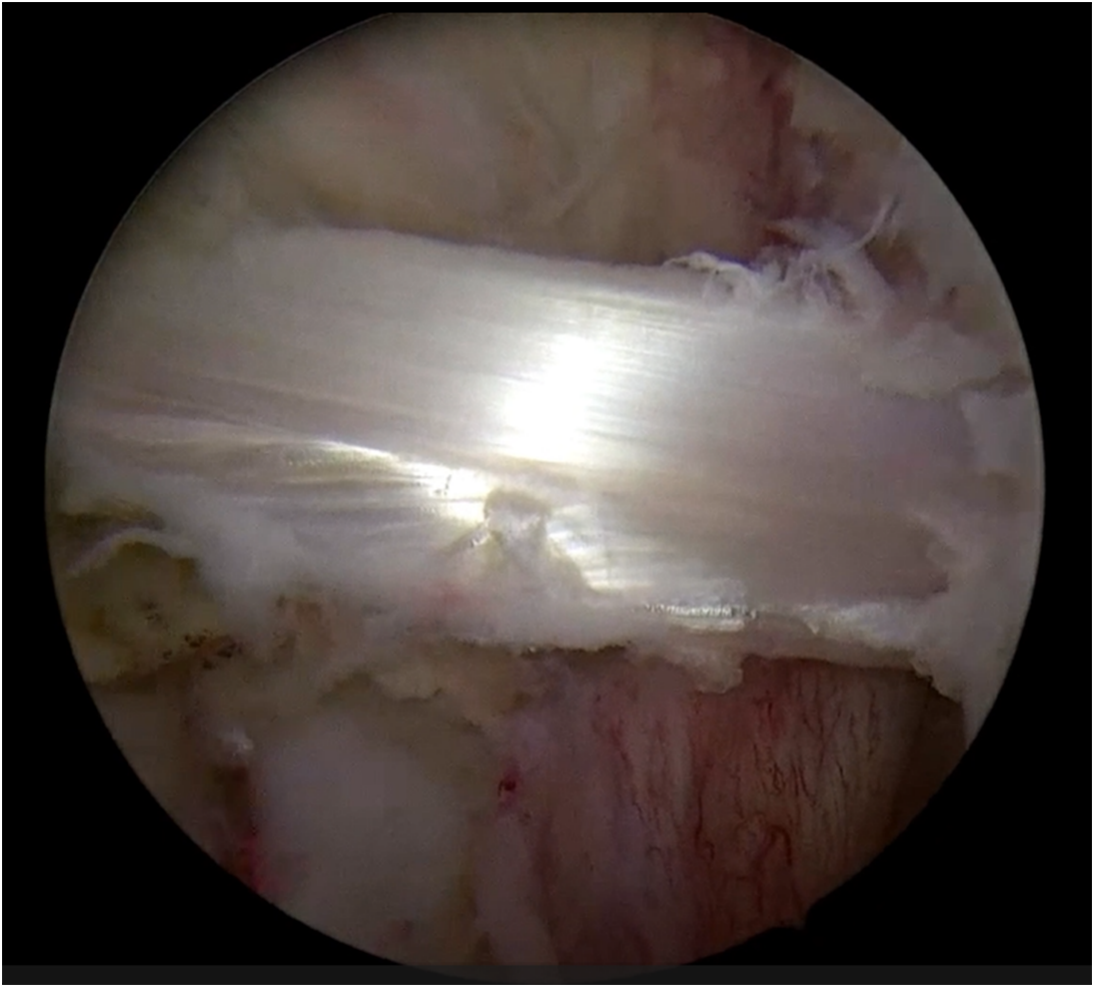
Patient in the beach‐chair position, posterior portal view in the left shoulder showed the presence of the long head of the biceps tendon inserted into the glenoid.

The sutured LHBT is then introduced into the joint and advanced toward the glenoid. No additional suture is used to pull the tendon through the tunnel via the subscapularis split; instead, gentle traction is applied directly to the suture strand, allowing the tendon to slide smoothly and seat securely within the predrilled glenoid tunnel, ensuring primary fixation stability (Figure 8).

With the tendon in place, external rotation of the upper limb is performed to verify a tension‐free course of the LHBT through the subscapularis and to identify any potential points of compression or overtensioning (Video 1).

After tendon fixation, the anteroinferolateral portal is used to reintroduce the cannula, and a superior labral repair is performed. A 1.5‐mm anchor (JuggerKnot, Zimmer Biomet, Warsaw, IN) is inserted at the superior glenoid rim to restore the anatomic footprint and reinforce the anterior stabilization construct (Table 1).

**TABLE 1:** Advantages and Disadvantages

Advantages	Disadvantages
Preserves anatomy and proprioception of the LHB	Risk of improper tunnel positioning
Provides dynamic stabilization (sling and hammock effect)	Potential mismatch between tunnel diameter and tendon size
Allows intraoperative decision‐making if labral tissue is insufficient	Limited working space in the anterior glenoid
Maintains external rotation range of motion	Risk of insufficient fixation if anchor placement is inadequate
Does not preclude future bone‐block procedures	
No specialized equipment is required; only a standard ACL instrumentation set, and suture anchors used for conventional capsulolabral repair are necessary, without increasing the overall procedural cost	

ACL, anterior cruciate ligament; LHB, long head of the biceps.

## DISCUSSION

In certain clinical scenarios where GBL is considered critical yet insufficient to warrant traditional bone‐block procedures such as the Latarjet or Bristow techniques which may be overly aggressive for the degree of bone loss and associated with a higher complication rate, DAS emerges as a viable alternative.

DAS may be particularly advantageous in athletes and high‐demand patients, offering a balance between biomechanical stability and soft‐tissue preservation.

The sling effect represents the primary stabilizing mechanism in both the Bristow and Latarjet procedures, accounting for approximately 51% to 77% of shoulder stabilization, depending on the position of the upper limb.^24^

DAS replicates these soft‐tissue benefits by combining the dynamic restraint provided by the LHBT with labral reconstruction as performed in the Bankart procedure, thereby establishing a triple soft‐tissue block to anterior translation (Table 2).

**TABLE 2:** How Can This Operation Go Wrong?

Proper posterior portal placement (slightly lateral, 2 cm inferior and 1 cm medial to posterolateral acromion), incorrect posterior portal placement compromising visualization
Use needle localization for precise anterolateral and anteroinferolateral portal placement. Poor portal positioning leads to suboptimal instrument trajectory
Create anteroinferolateral portal through subscapularis in line with humeral head equator. Injury to subscapularis because of improper portal creation
Perform LHB tenotomy at appropriate level (avoid proximal cuts). Cutting LHB too proximally, leading to diameter mismatch
Insert guide at 3‐o'clock position, flush with glenoid rim. Malposition tunnel compromising fixation and biomechanics
The tunnel diameter should match the LHB tendon size and should not exceed 5 mm, thereby preserving biceps tension and preventing Popeye deformity
Drill tunnel to approximately 20 mm depth, excessive drilling depth risking cortical breach
Impacted suture anchors commonly used in conventional capsulolabral repair are preferred, simplifying the procedure and reducing the risk of technical errors

LHB, long head of the biceps.

When combined with a Bankart repair, DAS not only enhances shoulder stability but also contributes to improved proprioception and retensioning of the inferior glenohumeral ligament. In Bristow procedures, medialization of the graft has been associated with increased recurrence rates of shoulder instability. Given that DAS replicates the biomechanical principles of the Bristow procedure, a more lateralized insertion point for the LHBT, as proposed in this study, may result in improved shoulder stability.^25^

Unlike bone‐blocking procedures, DAS avoids complications such as graft loosening, resorption, and pseudarthrosis.^26^

A similar triple soft‐tissue block mechanism was previously described using the conjoined tendon instead of the LHBT.^12^ However, because of its larger cross‐sectional area, this approach requires the creation of larger diameter bone tunnels to accommodate the interference screw.

Dynamic stabilization techniques employing interference screw fixation for the LHBT have also been reported. Nonetheless, this method may carry a higher risk of osteolysis and subsequent bone tunnel widening.^7^, ^27^

Another technique has been described using suture anchors through an onlay configuration.^23^ However, our approach follows the same principle as the button fixation technique,^21^, ^22^ in which a 20‐mm bone tunnel is drilled—matching the depth described for the button method. We believe this technique enhances tendon‐to‐bone contact, thereby promoting more effective biological healing and a more anatomic reconstruction.

Moreover, placing the suture anchor within this bone tunnel improves the mechanical strength of the fixation without compromising tendon vascularity, as it allows for adequate tensioning of the LHBT. This enhanced tensioning contributes to improved healing and provides superior stabilization, particularly in positions of abduction and external rotation. This approach also increases intraoperative flexibility, as it uses the same implants and instrumentation employed in Bankart repairs, without additional cost or unanticipated intraoperative challenges. In cases where labral degeneration or hypotrophy is encountered—conditions that may compromise the effectiveness of a Bankart repair—this technique offers a viable alternative without the need to convert to a bone‐block procedure intraoperatively.

Another advantage is that, in the event of failure, Bristow or Latarjet procedures may still be performed as revision options, since DAS does not compromise or preclude future bone augmentation strategies.

As potential disadvantages, there are theoretical risks of LHBT rupture or pain, glenoid fracture, suprascapular nerve injury, and cyst formation; however, such complications have not yet been reported in the literature.^22^, ^23^ Still, these issues may arise with longer term follow‐up.

This procedure is not recommended in cases of GBL greater than 20%,^17^ as biomechanical studies have showed excessive posterior translation of the humeral head in positions of abduction and external rotation. Additionally, DAS should be avoided in cases of pathological LHBT, given the risk of intraoperative or postoperative tendon rupture.

Surgical time is typically longer when compared with the standard arthroscopic Bankart repair, and the procedure also involves a steeper learning curve, particularly during arthroscopic training.^21^ However, by the end of the learning phase, the average surgical time was approximately 30 to 40 minutes.

In the authors’ opinion, this technique—as well as other similar procedures^11^—may represent a viable solution within the gray zone between Bankart repair and Bristow or Latarjet procedures,^17^ offering a balance between soft‐tissue preservation and enhanced anterior shoulder stability.

DAS with inlay fixation of the LHB offers a soft‐tissue‐based alternative for treating anterior shoulder instability with GBL of less than 20%, presenting with chronic shoulder dislocation associated with labral degeneration and concomitant SLAP lesions and especially beneficial for athletes with less than 5% GBL.

## DISCLOSURES

The authors (R.M., M.M., J.C.G., A.C., M.B., Á.M., H.L.) declare that they have no known competing financial interests or personal relationships that could have appeared to influence the work reported in this paper.
